# Clinical outcomes with second injection after insufficient bone cement distribution in unilateral kyphoplasty for osteoporotic vertebral compressive fracture: a cohort retrospective study

**DOI:** 10.1186/s13018-023-03968-2

**Published:** 2023-07-25

**Authors:** Youdi Xue, Jianwei Zhang, Zhaochuan Zhang, Weixiang Dai, Chao Ma

**Affiliations:** Department of Orthopedics, Xuzhou Central Hospital, Xuzhou Clinical School of Xuzhou Medical University, 199 Jiefang South Road, Xuzhou, 221009 Jiangsu Province People’s Republic of China

**Keywords:** Bone cement distribution, Cemented vertebra re-collapse, Osteoporotic vertebral compression fracture, Percutaneous kyphoplasty, Second injection

## Abstract

**Background:**

Bone cement distribution is an important factor affecting pain relief and long-term prognosis of osteoporotic vertebral compression fracture (OVCF) treated with vertebral augmentation. Unilateral percutaneous kyphoplasty (PKP) is the most common procedure, and insufficient bone cement distribution is more common than bilateral PKP. However, effective remedies are remain lack. In this study, sufficient cement distribution was achieved by adjusting the working channel followed by second cement injection as a remedy in cases with insufficient cement distribution, and the purpose was to evaluate the clinical outcomes by a retrospective cohort study.

**Methods:**

From July 1, 2017 to July 31, 2020, OVCF patients treated with unilateral PKP were included in this retrospective cohort study. According to the bone cement distribution (insufficient cement distribution was confirmed when the cement did not exceed the mid line of the vertebral body in frontal film or/and the cement did not contact the upper/lower vertebral endplates in the lateral film.) and whether second injection was performed during surgery, the patients were divided into three groups. Insufficient group: patients with insufficient cement distribution confirmed by fluoroscopy or postoperative x-ray. Second injection group: patients with insufficient cement distribution was found during the procedure, and second injection was performed to improve the cement distribution. Control group: patients with sufficient cement distribution in one injection. The Primary outcome was cemented vertebrae re-collapse rate. The secondary outcomes included operative time, radiation exposure, cement leakage rate, VAS, ODI, and adjacent vertebral fracture rate.

**Results:**

There are 34 cases in insufficient group, 45 cases in second injection group, and 241 cases in control group. There was no significant difference in baseline data and follow-up time among the three groups. Primary outcome: The injured vertebrae re-collapse rate of insufficient group was significantly higher than that of second injection group (42.22% vs 20.59%, *P* = 0.000) and control group (42.22% vs. 18.26%, *P* = 0.000). Kaplan–Meier survival analysis showed that there was no significant difference in the survival time between second injection group and control group (*P* = 0.741, Log-rank test), both of which were significant less than that in insufficient group (*P* = 0.032 and 0.000, respectively). Secondary outcomes: There was no significant difference in VAS score and ODI after operation between second injection group and control group, both of which were superior to those in insufficient group (*P* = 0.000). At the final follow-up, there was no significant difference in VAS and ODI among the three groups (*P* > 0.05). The operation time of second injection group was significantly higher than that of insufficient group (53.41 ± 8.85 vs 44.18 ± 7.41, *P* = 0.000) and control group (53.41 ± 8.85 vs 44.28 ± 7.22, *P* = 0.000). The radiation exposure of the second injection group was significantly higher than that of insufficient group (40.09 ± 8.39 vs 30.38 ± 6.87, *P* = 0.000) and control group (40.09 ± 8.39 vs 31.31 ± 6.49, *P* = 0.000). The cement leakage rate of second injection group (20.59%) was comparable with that of insufficient group (24.44%) and control group (21.26%) (*P* = 0.877). The length of hospital stay of the second injection group (4.38 ± 1.72) was comparable with that of insufficient group (4.18 ± 1.60) and control group (4.52 ± 1.46) (*P* = 0.431).

**Conclusions:**

When cement distribution is insufficient during unilateral PKP, second injection may relieve early pain, reduce the incidence of cemented vertebral re-collapse and adjacent vertebral fracture, without increasing the cement leakage rate, although this procedure may increase the operation time and radiation exposure.

## Introduction

Osteoporotic vertebral compression fracture (OVCF) is the most common complication of osteoporosis, which seriously affect the quality of life and daily activities, and even endanger life [1]. Surgical treatment is required for severe refractory pain and those who do not respond to conservative treatment. Vertebral augmentation (VA) such as percutaneous vertebroplasty (PVP) and percutaneous kyphoplasty (PKP) can stabilize the injured vertebra, relieve pain, and promote early ambulation. However some patients still have residual pain, and complications, such as re-collapse of the injured vertebra and adjacent vertebral fractures after VA [2]. Current studies suggest that the configuration, volume, and distribution of bone cement all are factors affecting pain relief and long-term prognosis [3]. Adequate bone cement distribution can fill the fracture gap and effectively relieve intractable pain. Liebschner et al. [4] found that asymmetric distribution of bone cement may lead to uneven stress conduction and trabecular micromotion, resulting in residual pain. In addition, bone cement close to the endplates can maintain the vertebral height and reduce the occurrence of injured vertebra re-collapse.

At present, unilateral PKP is the most common VA procedure for OVCF. Choosing the appropriate puncture location and direction can obtain cement distribution similar to bilateral procedure while reducing the surgical trauma and radiation exposure, shortening the operation time, and increasing the patient’s tolerance [5]. However, there remain some cases in which satisfactory cement distribution cannot be obtained [6]. Many studies have explored different approaches to improve cement distribution, such as using side opening trocar [7] and curved injection technique [8], but most spine surgeons are not familiar with these new devices and the cost is higher than common procedures. Some authors [9] have adopted contralateral supplementary injection to improve cement distribution, but this will increase surgical trauma and radiation exposure. In this study, sufficient cement distribution was achieved by adjusting the working channel followed by second cement injection as a remedy in cases with insufficient cement distribution, and the clinical outcomes was evaluated by a retrospective cohort study.

## Methods

### Study design and patient selection

This study followed the ethical principles outlined in the World Medical Association Declaration of Helsinki and was approved by the Ethics Committee of Xuzhou Central Hospital, written informed consent was obtained at the final follow-up. This retrospective cohort study consecutively included OVCF patients who presented to Xuzhou Central Hospital (A tertiary hospital) and underwent VA from July 1, 2017 to July 31, 2020, the study data were derived from the patients’ medical records and imaging data. Inclusion criteria included: Aged over 60 years, T score based on dual-energy X-ray absorptiometry (DEXA) was lower than − 2.5, single-level OVCF, without neurological symptoms, unilateral PKP was performed, the follow-up duration was more than 24 months. Exclusion criteria included: Non-osteoporotic vertebral fracture caused by tumour, inflammation, violence, etc.; multilevel vertebral fractures; PVP or bilateral PKP was performed, the previous history of surgical trauma in injured vertebra or adjacent vertebra, incomplete clinical and imaging data.

### The definition of insufficient cement distribution

In this study, unilateral PKP was performed in single-level OVCF, and when cement leakage outside the vertebral body or reach the posterior vertebral wall, the injection was suspended for about 1–2 min, and resumed again until x-ray revealed that the cement still had continuous leakage or contact with the spinal canal. Under this condition, insufficient cement distribution was defined when the frontal x-ray showed that the cement did not exceed the mid line of the vertebral body or/and the cement did not contact the upper/lower vertebral endplate in the lateral x-ray.

### Surgical procedures

The operation was performed independently by three senior doctors (Youdi Xue, Zhaochuan Zhang, and Weixiang Dai) who had performed more than 200 vertebral VA, and the procedure was similar to a previous study [10]. All the procedures in this study use the same instruments (Shandong Dragon Crown Medical Supplies Inc., China.) and bone cement (Heraeus Medical GmbH, Germany). The patient was placed in a prone position, keep the abdomen empty and fracture site hypertension. Fluoroscopic localization was performed to determine the injured vertebra, the distance between the skin puncture point and the midline was measured according to preoperative CT or MRI, the junction between the superior facet and transverse process base was used as the entry point. When the lateral film showed the trocar reach the anterior third of the vertebral body, the frontal film showed that the trocar should located or crossed the centre vertebral line, then implanted and expanded the balloon, injected the bone cement after removing the balloon.

### Second injection

When the insufficient cement distribution was confirmed, retracted the working channel to the posterior edge of the vertebral body, and changed the position and direction of the working channel to the area lack of cement distribution, then injected the cement to obtain sufficient cement distribution in the vertebra.

### Postoperative treatment

Ambulation was allowed 6 h after surgery, trunk extensor exercise and anti-osteoporosis drugs were prescribed. Outpatient and/or telephone follow-up was performed every three months after surgery, and if low back pain recurrence, MRI was performed to identify adjacent vertebral fracture or cemented vertebral re-collapse.

### Patients group

According to the bone cement distribution and whether second injection was performed during surgery, the patients were divided into three groups. Insufficient group: patients with insufficient cement distribution confirmed by fluoroscopy or postoperative x-ray. Second injection group: patients with insufficient cement distribution during the procedure, and second injection was performed to improve the cement distribution. Control group: patients with sufficient cement distribution in one injection.

### Data collection

The sex, age, body mass index (BMI), bone mineral density (BMD)-T score, fracture location, history of injury, smoking status, visual analogue scales (VAS) for low back pain, and the Oswestry Disability Index (ODI) were recorded. Bone mineral density at the femoral neck and lumbar spine was measured using dual-energy X-ray absorptiometry (DEXA) to evaluate the degree of osteoporosis. According to the fracture location, thoracic segment (T5-T10), thoracolumbar segment (T11-L2), and lumbar segment (L3-L5) was divided. VAS score: 0 represents no pain, 1–3 points, indicating mild pain, 4–6 points, more obvious pain, 7–10 points, very severe and unbearable pain. ODI score: comprehensive assessment of activity of daily living before and after surgery and during follow-up is performed using ODI, consisting of 10 questions, 6 options for each question, with the first option selected having a score of 0 and the last option having a score of 5, total score = (score obtained/the number of questions answered multiplied by 5) * 100%, with higher scores indicating more severe dysfunction.

The primary outcome was the injured vertebra re-collapse rate. According to the criteria established in previous literature [11, 12], the injured vertebra re-collapse was confirmed when one of the following two conditions emerged during follow-up: 1. Compared with postoperative, the injured vertebra re-collapse rate was more than 15%. The formula for calculating the injured vertebra re-collapse rate: anterior vertebral height after the operation subtract anterior vertebral height at the last follow-up/anterior vertebral height after the operation * 100%. 2. Compared with postoperative, local kyphosis angle (LKA) was increased by 10 degree. Because OVCF is associated with endplate disc complex injury frequently, disc space narrowing and intervertebral angle reduction may occur with time, measurement of kyphotic angles including adjacent vertebra and disc space may falsely elevated, so we measure the angle formed by the upper endplate and the lower endplate of the injured vertebra as LKA.

Secondary outcomes included operative time, radiation exposure, VAS, ODI, cement leakage rate, and adjacent vertebral fracture rate (New onset of low back pain during follow-up and MRI showed high signal intensity in the T2 and fat-suppressed phases of the adjacent vertebra, suggesting adjacent vertebral fracture).

### Statistical analysis

Statistical analysis was performed using SPSS 26.0(SPSS, Inc., USA). The continuous variables were expressed as the mean and the standard deviation, the categorical variables were expressed as numbers and frequencies. Two-independent sample t-test was used for comparison between groups, the LSD-t test was used for multiple comparisons, and paired sample t-test was used for comparison within groups. The Chi-square test or Fisher test was used for sex, cement leakage rate, the injured vertebra re-collapse rate, and adjacent vertebra fracture rate. The survival time was calculated from the beginning of surgery until the occurrence of the injured vertebra re-collapse during follow-up or until the censor date of June 30, 2022.

Kaplan–Meier survival analysis was used to calculate the injured vertebra re-collapse rate, and to describe the survival process, and the log-rank test was used to compare the differences in the survival time distributions among the three groups. Cox regression models were used to calculate hazard ratios (HR) and 95% confidence intervals (CI). At first, group (whether the second injection was performed) was included as an independent factor in the model to calculate unadjusted HR; then sex, age, bone mineral density, and fracture location were included in the model to calculate adjusted HR. When *P* < 0.05, differences were considered statistically significant.

## Results

**Fig. 1:**
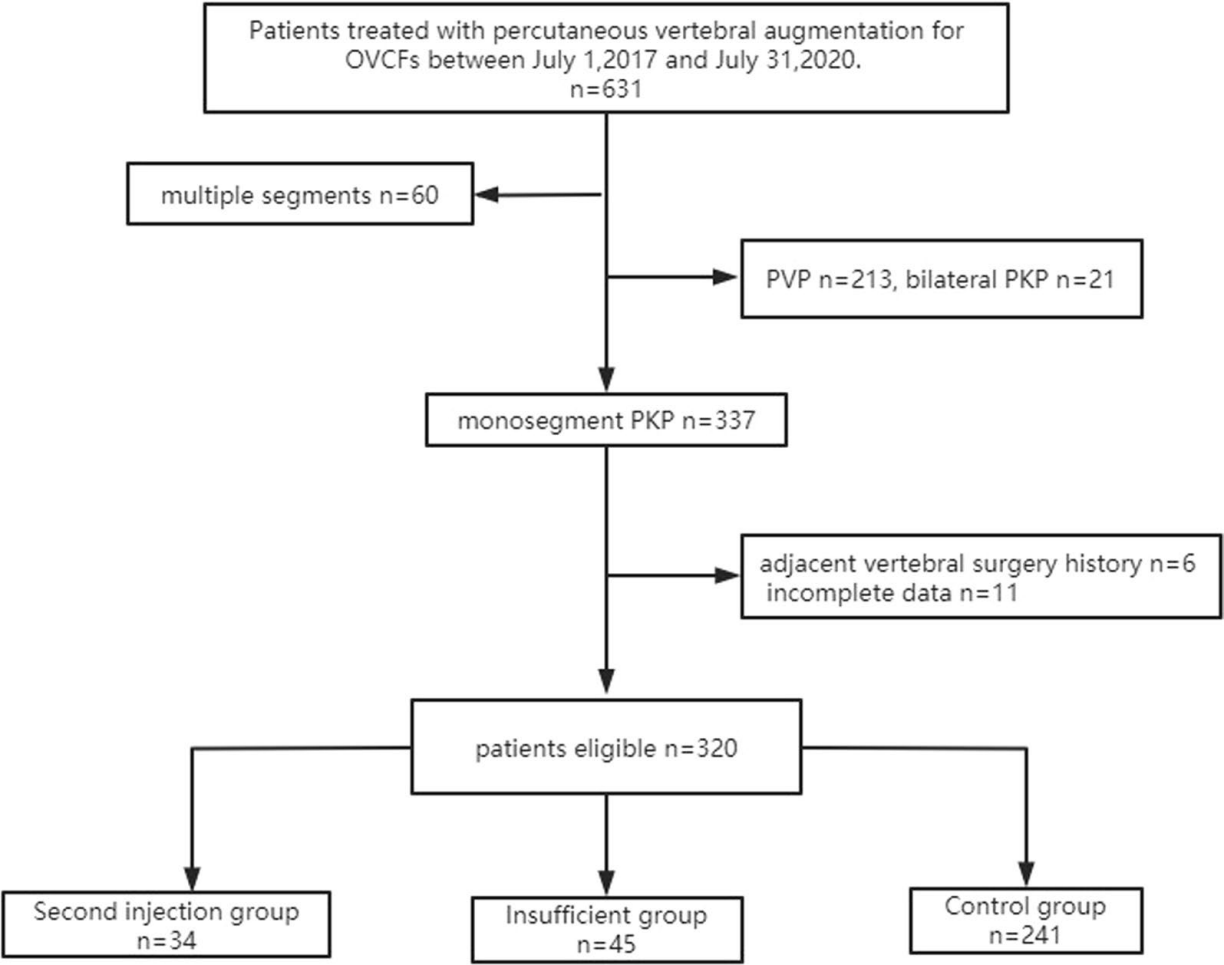
Flowchart of patients selection

**Fig. 2:**
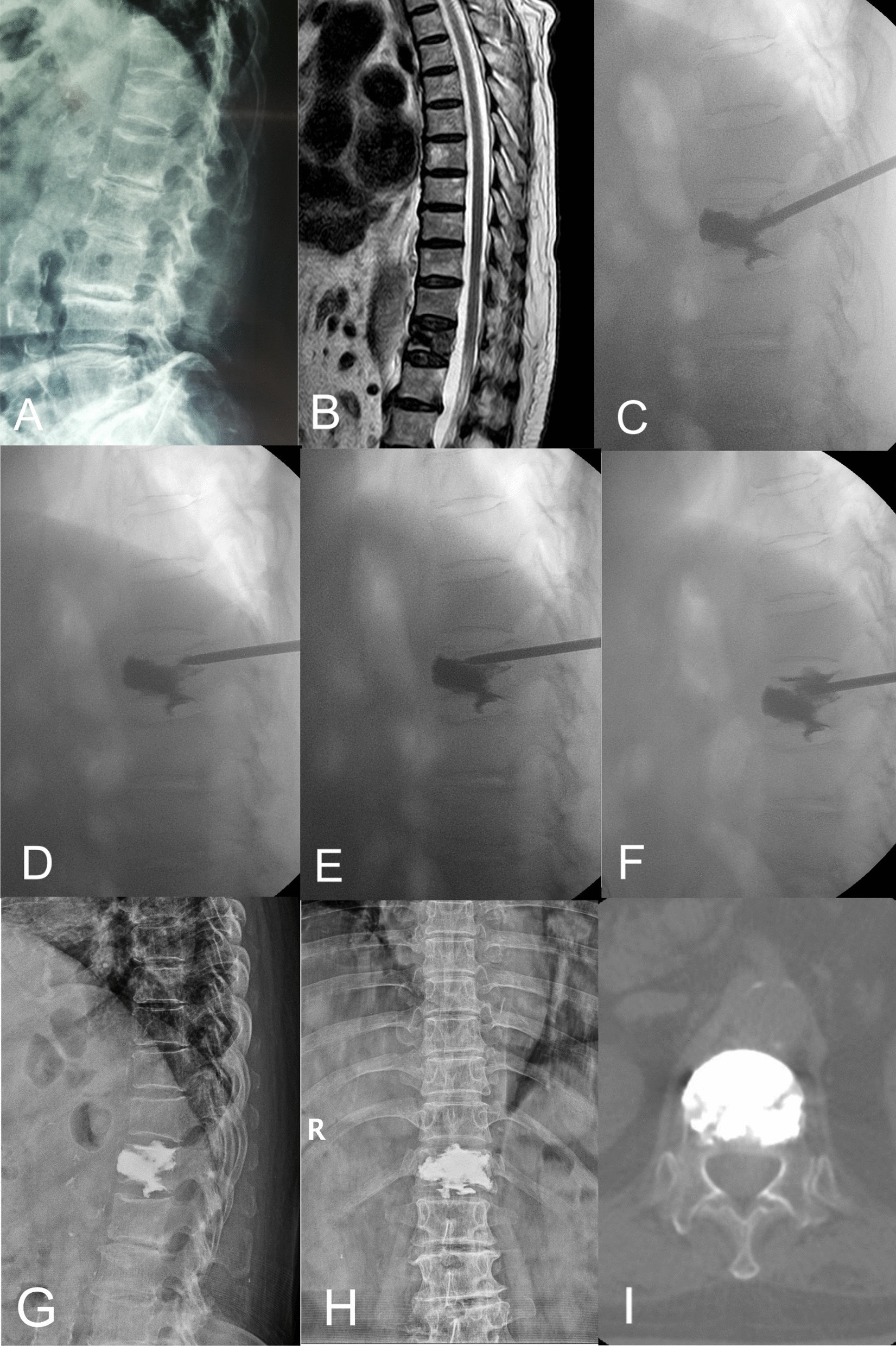
A 71-year-old female patient diagnosis as T12 OVCF was treated with unilateral PKP. **A**, **B** Preoperative X-ray and thoracic MRI showed fresh compressive fracture at T12. **C** Intraoperative fluoroscopy showed insufficient bone cement distribution at the upper part with cement leakage in the lower disc space. **D**, **E** Working channel was retracted to the posterior wall of the vertebra, and put the working channel to the area lack of cement distribution. **F** After second injection, sufficient cement distribution was obtained. **G**–**I** Postoperative X-ray and CT showed sufficient bone cement in T12 vertebral body

A total of 631 OVCF patients who underwent VA from July 1, 2017 to July 31, 2020, were admitted to the hospital, excluding multilevel OVCFs (*n* = 60), PVP surgery (*n* = 213), bilateral PKP surgery (*n* = 21), previous adjacent vertebral surgery (*n* = 6) and incomplete clinical and imaging data (*n* = 11), finally a total of 320 patients were included in this study. According to the bone cement distribution and whether the second injection was performed during surgery. The patients were divided into three groups. Insufficient group: 45 cases of insufficient cement distribution were confirmed by fluoroscopy or postoperative x-ray. Second injection group: 34 cases, insufficient cement distribution was found during the operation, and second injection was performed to improve the cement distribution. Two hundred forty-one cases achieving adequate cement distribution in one injection were recognized as the control group (Fig. 1). There was no significant difference in baseline data and follow-up time among the three groups (Table 1). The typical case in second injection case is showed in Fig. 2.

**Table 1:** Summary of baseline data of the three groups

Characteristic	Insufficient group *n* = 45	Second injection group *n* = 34	Control group *n* = 241	*P* value
Age	71.67 ± 6.16	70.61 ± 7.05	73.57 ± 8.44	0.085
Sex				0.822
Female—*n* (%)	33(73.33)	24(70.59)	166(68.88)	
Male—*n* (%)	12(26.67)	10(29.41)	75(31.12)	
Bone mineral density (T-score)	− 3.26 ± 0.43	− 3.35 ± 0.42	− 3.16 ± 0.43	0.379
Body mass index (kg/m^2^)	23.41 ± 2.94	22.16 ± 2.99	22.47 ± 2.90	0.096
Injury history—*n* (%)	18(40)	13(38.24)	103(42.74)	0.859
Smoking—*n* (%)	6(13.33)	6(17.65)	40(16.60)	0.874
Fractured location				0.769
Thoracic segment (T5–T10)	8(17.78)	5(14.71)	27(11.20)	
Thoracolumbar segment (T11**–**L2)	31(68.89)	24(70.59)	183(75.93)	
Lumbar segment (L3**–**L5)	6(13.33)	5(14.71)	31(12.86)	
Follow-up duration (months)	44.22 ± 8.44	43.97 ± 10.85	41.56 ± 9.09	0.095

**Fig. 3:**
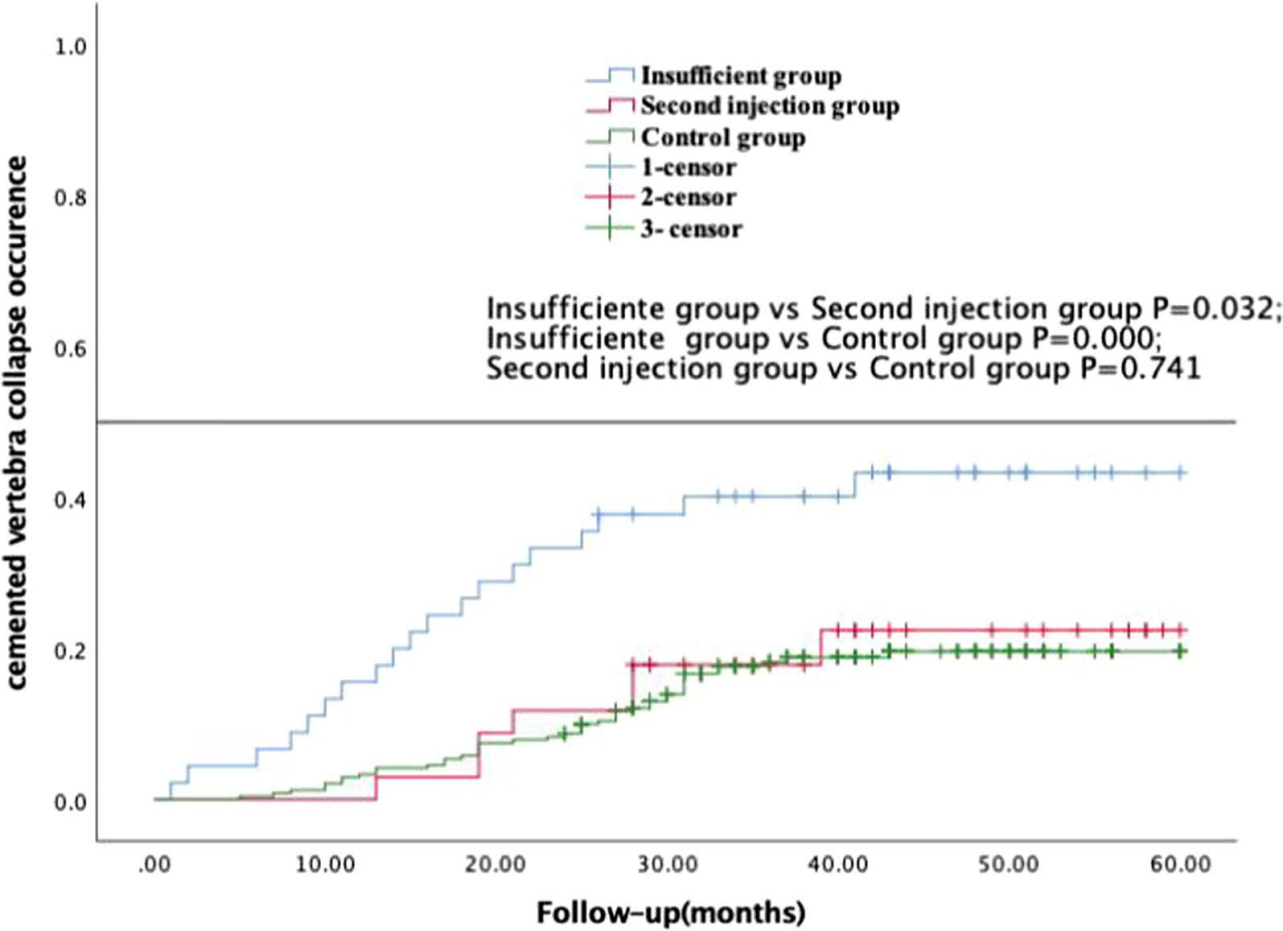
Kaplan–Meier survival curve of the survival time to the cemented vertebra re-collapse among three groups

Primary outcome: During the final follow-up, 19 patients in insufficient group developed injured vertebra re-collapse (42.22%), with a mean survival time of 41.26 ± 3.37, 95% CI: 34.69–47.89, 7 patients in the second injection group developed injured vertebra re-collapse (20.59%), with a mean survival time of 52.15 ± 2.67, 95% CI: 46.93–57.38, and 44 patients in the control group developed injured vertebra re-collapse (18.26%), with a mean survival time of 52.89 ± 0.99, 95% CI: 50.96–54.82 (Table 2). Kaplan–Meier survival analysis showed that there was no significant difference in the survival time between the second injection group and control group (*P* = 0.741, Log-rank test), both of which were significant less than that in the insufficient group, (*P* = 0.032 and 0.000, respectively) (Fig. 3). Using the group as the only variable, the cox regression analysis showed that the second injection was associated with a significantly reduced injured vertebra re-collapsed incidence (HR = 0.401, 95% CI = 0.168–0.955, *P* = 0.039), then added sex, age, bone mineral density, and fracture location in the model, the results showed that second injection (HR = 0.386, 95% CI = 0.161–0.921, *P* = 0.032) and bone mineral density (HR = 0.314, 95% CI = 0.118–0.839, *P* = 0.021) were associated with a significantly reduced vertebra re-collapsed rate (Table 3).

**Table 2:** The injured vertebrae re-collapse rate and survival time of the three groups at the final follow-up

Characteristic	Insufficient group *n* = 45	Second injection group *n* = 34	Control group *n* = 241	*P* value
Injured vertebra re-collapse rate (%)	42.22^#^	20.59	18.26	0.000
Survival time (month)	41.26 ± 3.37^#^	52.15 ± 2.67	52.89 ± 0.99	0.000
95% CI	34.69–47.89	46.93–57.38	50.96–54.82	–

^#^Compared with the other two groups, *P* = 0.000, *CI* confidence interval

**Table 3:** Multivariate Cox regression model for the survival time to the injured vertebrae re-collapse

Variable	HR (95% Cl)	*P* value
Model 1		
Group	0.401 (0.168–0.955)	0.039
Model 2		
Group	0.386 (0.161–0.921)	0.032
Sex	1.104 (0.461–2.641)	0.825
Age	1.001 (0.944–1.062)	0.964
Bone mineral density	3.183 (1.192–8.504)	0.021
Fractured location	1.645 (0.731–3.701)	0.229

*HR*—Hazard ratio, *CI*—confidence interval

Secondary outcomes: The VAS and ODI after the operation in the three groups were significantly improved compared with those before the operation (*P* < 0.05). There was no significant difference in VAS score and ODI after operation between the Adequate group and the Control group, both of which were superior to those in the Inadequate group (*P* = 0.000). During the final follow-up, there was no significant difference in VAS and ODI among the three groups (*P* > 0.05) (Table 4). The operation time of the second injection group was significantly higher than that of insufficient group (53.41 ± 8.85 vs 44.18 ± 7.41, *P* = 0.000) and control group (53.41 ± 8.85 vs 44.28 ± 7.22, *P* = 0.000). The radiation exposure of the second injection group was significantly higher than that of the insufficient group (40.09 ± 8.39 vs 30.38 ± 6.87, *P* = 0.000) and control group (40.09 ± 8.39 vs 31.31 ± 6.49, *P* = 0.000). The cement leakage rate of the second injection group (20.59%, 7/34) was comparable with that of the insufficient group (24.44%, 11/45) and control group (21.26%, 51/241) (*P* = 0.877). The hospital stay of the second injection group (4.38 ± 1.72) was comparable with that of the insufficient group (4.18 ± 1.60) and control group (4.52 ± 1.46) (*P* = 0.431) (Table 5).

**Table 4:** The clinical outcomes between the three groups preoperative, postoperative and at the final follow-up

Characteristic	Insufficient group *n* = 45	Second injection group *n* = 34	Control group *n* = 241	P value
VAS				
Preoperative	6.44 ± 1.41	6.85 ± 1.31	6.58 ± 1.28	0.372
Postperative	2.53 ± 1.18^#^	1.91 ± 1.00	1.75 ± 0.97	0.000
Final follow-up	1.40 ± 0.94	1.44 ± 0.82	1.16 ± 0.82	0.058
ODI				
Preoperative	62.76 ± 13.94	65.47 ± 8.79	62.00 ± 13.75	0.363
Postperative	33.24 ± 9.14^#^	24.26 ± 6.42	25.08 ± 7.80	0.000
Final follow-up	17.33 ± 4.12	16.53 ± 3.99	18.31 ± 6.14	0.168

^#^Compared with the other two groups, *P* = 0.000

**Table 5:** The procedure-related data in the three groups during their duration of hospital stay

Characteristic	Insufficient group *n* = 45	Second injection group *n* = 34	Control group *n* = 241	*P* value
Operation time (min)	44.18 ± 7.41	53.41 ± 8.85*	44.28 ± 7.22	0.000
Radiation exposures	30.38 ± 6.87	40.09 ± 8.39*	31.31 ± 6.49	0.000
Cement leakage—*n* (%)	11(24.44)	7(20.59)	51(21.26)	0.877
Stay of hospital (days)	4.38 ± 1.72	4.18 ± 1.60	4.52 ± 1.46	0.431
Adjacent vertebral fracture—*n* (%)	11(24.44)*	4(11.76)	23(9.54)	0.018

^*^Compared with the other two groups, *P* < 0.05

## Discussion

Our study showed that in cases with poor cement distribution during the unilateral PKP for OVCF, second cement injection may improve cement distribution, obtain similar clinical results as the control group, and do not increase the cement leakage rate, but increase the operation time and radiation exposure. Compared with insufficient group, it can effectively relieve pain, improve mobility, and reduce the incidence of injured vertebra re-collapse and adjacent vertebral fracture.

In this study, the primary outcome showed that the injured vertebra re-collapse rate was similar in the second injection (20.59%) and the control group (18.26%), which were both lower than those in insufficient group (42.22%), these results were consistent with the literature [13]. Previous study showed that the injured vertebrae re-collapse rate is not low, and it will significantly affect the clinical outcomes [14]. Some studies found that excessive restoration of vertebral height is associated with injured vertebra re-collapse [2], and others found that inadequate distribution of bone cement, especially lack of cement close to the endplate is the main cause of re-collapse [15, 16]. Sufficient bone cement distribution to the both endplates can increase the strength of the vertebral body about 11 times, thus reduce the risk of re-collapse after augmentation [17].

Unilateral PKP attempts to achieve bilateral symmetrical cement distribution through one puncture, thereby reducing surgical trauma, radiation exposure, and operation time. Selecting the right puncture location and direction is the key to obtain satisfactory distribution of bone cement. In some cases, although the direction and position of the puncture channel are satisfactory, it may also lead to insufficient bone cement distribution due to other reasons, such as fractured areas [11] and cement leakage [18]. If the posterior wall defects is present, bone cement may leak into the spinal canal or intervertebral foramen through the defect area. Additionally, bone cement may also leak through the vein, and serious complications such as pulmonary embolization may occur. When these leakage happened, injection needs to be stopped at once even if a sufficient distribution is not achieved [19].

In this study, C-arm fluoroscopy was used to evaluate cement distribution in frontal and lateral films during the procedure, and to decide whether it is necessary to perform second injection procedure. The second procedure should be performed immediately once insufficient cement distribution is identified. On the one hand, it can reduce the complications result from cement leakage, on the other hand, it can avoid cement solidification in the vertebral body result from waiting too long, so the secondary injected cement cannot mix together with the former one.

The procedure in this study was similar to the one introduced by Chen et al. [20], with no need for additional instrumentation, and is easy to perform without increasing the cement leakage and other complications rates. In the insufficient group, the VAS and ODI after operation were significantly higher than those in second injection group and control group. At the last follow-up, there was no significant difference in VAS and ODI among the three groups, presumably related to complete fracture healing, which was consistent with the previous study [20, 21]. The analgesic mechanism of vertebral augmentation in OVCF has not been fully clarified, most researchers believe that it is related to nerve endings inactivation by high temperature [22] and trabecular stabilization after cement solidification [23]. It has been reported that there are many factors affecting the effect of vertebral augmentation, including bone mineral density, multilevel fractures, and disease duration [24]. In recent years, studies on the factors such as cement volume, shape, and distribution have been increased. Adequate bone cement distribution in the fracture area is considered to be the main factor in ensuring the surgical effect [4, 25]. If the cement cannot spread sufficiently into the fracture area, the fractured trabeculae cannot be fixed, so the pain due to trabecular micromotion cannot be relieved [26]. Furthermore, because cement strength is significantly higher than that of cancellous bone, insufficient cement distribution can lead to local stress imbalance, which is harmful to the spinal function recovery. Repeated procedures have been performed in patients with persistent pain or new pain after insufficient bone cement distribution, and satisfactory pain relief and vertebral height recovery was obtained, further confirming that pain may be related to cement deficiency in fracture area or cemented vertebra re-collapse [14, 27, 28].

The adjacent vertebral fracture rate after VA is as high as 12–52% [29], because most adjacent vertebral fractures occur within 3 months after surgery and are related intervertebral cement leakage, many studies suggest that stress variety caused by cement implantation may be the main causes [29]. Further studies have found that cement distribution characteristics, high cement volume, and insufficient cement distribution [30] are all risk factors for adjacent fracture. Tanigawa et al. [31] divided the cement distribution into spongy and mass-like types, and no significant difference was found between the two distributions in pain relief, but the adjacent vertebral fracture rate with mass-like distribution was significantly higher. Polikeit et al. [32] found that implanted overdose bone cement may induce higher stiffness of the injured vertebra and more stress on the adjacent vertebra, so the adjacent vertebra fracture may be easily happened. Similarly, because the elastic modulus and compressive strength of cement are much higher, insufficient cement distribution may lead to uneven stress transmitted to adjacent disc and vertebra, which may affects the fracture occurrence of the adjacent vertebra and cemented vertebra [33, 34], this is consistent with the results of this study. So far, the optimal amount of bone cement during vertebral augmentation remains controversial; however, we believe that excessive or insufficient bone cement implantation in the injured vertebra can both affect the adjacent vertebral fracture. This study introduced a novel, easy handle and safe procedure to improve cement distribution, and we believe this procedure will improve clinical outcome and long-term prognosis after unilateral PKP for OVCF.

### Limitations

1. This study is a retrospective cohort study, and the number of cases was small, so studies with higher levels of evidence and larger subjects, such as RCT studies, are needed to further confirm the effect of second injection;
2. The procedure was not completed by the same doctor, which may affect the study results due to different experiences and skills by the surgeons;
3. Anti-osteoporosis treatment is an important factor for the operation results, but the anti-osteoporosis drugs used in this study were not uniform, which may have an impact on the results of the study;
4. VA techniques have various procedures, but this study only analysed unilateral PKP, which lacked comparison with other procedures.

In conclusion, when cement distribution is insufficient during unilateral PKP, second injection can improve the cement distribution in the injured vertebra, although this will increase the operation time and radiation exposure, it may relieve pain, reduce the incidence of cemented vertebral re-collapse and adjacent vertebral fracture, without increasing the cement leakage rate.

## Data Availability

Not applicable.
